# Under pressure: a call to increase access to spinal manometers as a critical component of the management of cryptococcal meningitis

**DOI:** 10.1097/QAD.0000000000004545

**Published:** 2026-07-30

**Authors:** Jonathan Falconer, Jack Adams, Sile Molloy, Kennedy Mupeli, Nelesh P. Govender, Joseph N. Jarvis, Thomas S. Harrison, David S. Lawrence

**Affiliations:** a School of Pathology, Faculty of Health Sciences, University of the Witwatersrand, Johannesburg; b South Africa Medical Unit, Médecins Sans Frontières, Cape Town, South Africa; c City St George's, University of London; d Clinical Research Department, Faculty of Infectious and Tropical Diseases, London School of Hygiene and Tropical Medicine, London, UK; e Botswana Harvard Health Partnership, Gaborone, Botswana.

**Keywords:** advanced HIV disease, cryptococcal meningitis, global health, health economics, HIV, low-resource settings

## Introduction

*Cryptococcus* remains the most common cause of adult meningitis in Africa and the second leading cause of AIDS-related mortality after tuberculosis [1,2]. The past 20 years have seen significant progress in the treatment of HIV-associated cryptococcal meningitis: shorter amphotericin B-based induction treatments in combination with flucytosine and fluconazole have resulted in a substantial survival benefit[3,4]. As a result, access to, and implementation of, these regimens has been a focus for successful advocacy efforts [5,6]. In addition to effective antifungal therapy, management of raised intracranial pressure (ICP), a common complication of cryptococcal meningitis, is a core component of care included in the World Health Organization (WHO) guidelines on the management of cryptococcal disease in people living with HIV [7].

Effective ICP management requires a complex interplay of available resources, healthcare worker capacity and training, structured protocols, and frequent clinical assessment which is often challenging to achieve, particularly in low-resource settings. A critical component of management is spinal manometers, which are the preferred bedside method for measuring ICP. Aside from a few centers mainly in South Africa, spinal manometers are not widely available, often due to perceived challenges with cost and access. In the absence of manometers, ICP frequently goes unmeasured. Alternative options for assessing cerebrospinal fluid (CSF) pressure include estimating pressure based on the CSF drip rate from the lumbar puncture (LP) needle [8], or using intravenous (i.v.) infusion lines attached to the spinal needle as a makeshift alternative. Despite this, the evidence supporting the use of i.v. infusion lines for ICP measurement is inconclusive. This opinion aims to summarize the literature around the benefits of effectively managing raised ICP, discuss whether i.v. infusion lines can serve as an acceptable alternative to spinal manometers, and explore the potential economic impact of broadening access to manometers in low-resource settings.

### The importance of raised intracranial pressure management

Raised ICP in cryptococcal meningitis is associated with a high fungal burden in the CSF and poor clinical outcomes [9]. It is hypothesized that raised ICP occurs due to fungal cells and capsular polysaccharide obstructing reabsorption of CSF from the arachnoid villi [10]. Historically, 65–93% of acute deaths from cryptococcal meningitis have occurred in patients with elevated ICP at baseline [11,12]. In contemporary routine care in low-resource settings, where optimal ICP management is often challenging, these mortality differences are still observed based on baseline ICP. A 2016 South African study demonstrated that patients who received adequate ICP management during their inpatient stay had significantly lower in-hospital mortality compared to those who did not (12% [5/43] vs. 29% [18/62]; *P* = 0.03) [13].

In well-resourced clinical trial settings, effective management of raised ICP through serial pressure monitoring and CSF drainage has been associated with improved survival. A pooled analysis of over 500 participants with cryptococcal meningitis from nine clinical trials conducted in Africa and Thailand found no increased mortality associated with raised baseline opening pressure when ICP was actively managed using manometers and standardized protocols [9]. The AMBITION-cm trial, which enrolled 844 participants, similarly showed no significant difference in mortality between those with raised baseline opening pressure and those with normal baseline pressure [4]. Two studies that have quantified the relative risk reduction suggest effective ICP management could reduce the relative risk of death by 20–69%, although selection and immortal time bias in the methodologies put these at risk of overestimating the impact [13,14].

It is important to distinguish between the availability of spinal manometers and effective intracranial pressure management programs. The impact of ICP management in clinical trials has been reported in settings where manometers were embedded within structured protocols, regular pressure monitoring, and assessment by experienced staff. These findings do not isolate the independent effect of the manometer but suggest that accurate measurement and structured ICP management are critical components of effective care. Spinal manometers should therefore be viewed as a necessary component of care, enabling accurate measurement to guide therapeutic CSF drainage, but need to be provided within appropriate systems, alongside training and institutional commitment.

### Barriers to effective intracranial pressure management

Barriers to the effective management of raised ICP in low-resource settings are largely driven by challenges in performing LPs when clinically indicated. Qualitative studies exploring the reasons for what has been termed the ‘tap gap’ have shown the reasons are multifactorial and occur at the person, healthcare provider and health system levels [15–17].

Among these, an important facilitator of provider uptake is access to equipment to perform the procedure, with access to appropriate instruments including manometers reported to be motivating factors [15,16]. This also includes access to lumbar puncture needles; in their absence, needles intended for other uses, such as for cannulation, are often used as substitutes [18]. This is one of several interconnected factors contributing to low lumbar puncture uptake. Other provider-level barriers include adequate staff training, particularly in the context of health systems with high staff turnover and low staff-to-patient ratios that make time consuming procedures like lumbar puncture a challenge.

In addition to provider-level barriers, patient refusal of lumbar puncture is also commonly reported [15]. This highlights the need to pair efforts to increase access and improve provider uptake with community engagement and education to address patient-level barriers.

## Discussion

### Is intracranial pressure measurement with an i.v. infusion line an acceptable surrogate for spinal manometers?

Access to lumbar puncture needles and spinal manometers is a key barrier in low-resource settings. A commonly used alternative is an i.v. infusion line connected to the spinal needle and held vertically, with CSF pressure estimated by measuring the height of the fluid column with a tape measure. It is unclear whether pressure measurements obtained using i.v. infusion lines are equivalent to those obtained with standard spinal manometers. The internal diameter of a spinal manometer is typically around 1 mm, whereas i.v. infusion lines have a larger bore of approximately 3 mm. According to Jurin's law [19], the height a fluid column achieves due to capillary action is inversely proportional to the tube's radius. Therefore, in theory, a given pressure could produce different fluid heights in tubes of different diameters. However, hydrostatic pressure is independent of tube diameter, and capillary effects become negligible in tubes wider than 1–2 mm. The net effect of these opposing forces – capillarity pulling fluid upward and gravity counteracting it – makes it difficult to predict with certainty the height difference expected between a 1 mm manometer and a 3 mm i.v. line.

Two studies have sought to empirically quantify the difference by directly comparing the two methods in practice. One study, conducted in Tanzania, measured ICP in 35 patients by comparing the opening pressure measurements taken using both a spinal manometer and an i.v. infusion line [20]. Measurements taken with the i.v. infusion line were highly correlated with those obtained using the manometer (*r*^2^ = 0.96). In this study, the manometer was used first to measure the opening pressure, followed by the i.v. infusion line, but no account was taken for CSF loss during the transition from the manometer to the infusion line. The measurement using the i.v. infusion line will therefore have been an underestimate. The primary aim of this study was to assess the impact of using i.v. infusion lines to manage elevated ICP. The authors observed a significant reduction in 10-week mortality, from 75% (48/64) in a historical comparison group to 46% (16/35) with the use of i.v. infusion lines. However, this comparison was made between a retrospective cohort and a prospective cohort treated with a new model of care extending beyond the use of i.v. infusion lines for ICP management, therefore introducing several biases.

A subsequent study sought to refine the methods of the Tanzanian study by using a 3-way stopcock to simultaneously connect a spinal needle to both a manometer and an i.v. infusion line [21]. This allowed a switch between the two measurement modalities without fluid loss. They measured opening pressure in 100 patients and found that the i.v. infusion line consistently underestimated ICP compared to the manometer, with a non-linear conversion between the two methods. They proposed a conversion equation: *manometer pressure = 0.85 × infusion line pressure + 8.9*. Therefore a measurement of 19 cm with an i.v. infusion line would be equivalent to the widely accepted threshold of 25 cm for elevated pressure when measured with a spinal manometer. Despite their study the authors concluded “*We still assert that measurement of CSF OP [opening pressure] should be performed by standard spinal manometry, a tried-and-tested method of measuring CSF OP. The practice of using the IVGS [intravenous giving set] to measure CSF OP should be discouraged, especially if no correction of the result is made.*” The WHO 2022 guidelines on cryptococcal care do not provide specific recommendations for interpreting pressure measurements obtained using i.v. infusion lines or a threshold for what constitutes ‘elevated’ ICP [7].

Potential underestimation of the numeric opening pressure carries clinical risks when protocols often quote fixed thresholds for clinical action. False reassurance about the ICP may lead to a delay or absence of therapeutic lumbar punctures as well as failing to identify patients at highest risk for neurologic deterioration. Conversely, over-drainage of CSF in patients without markedly elevated ICP is generally considered to carry lower clinical risk.

Where spinal manometers are unavailable, i.v. infusion lines may function as a temporary stopgap. In such circumstances a pragmatic interpretation of the data would suggest either prioritizing symptom-guided therapeutic lumbar punctures over numeric thresholds or applying conservative correction factors such as the one described above. Pressures above ~19 cm vertical height when measured with a standard i.v. infusion line may roughly correlate with the manometer derived 25 cm threshold according to the Mogambery *et al.* conversion factor [21].

In addition to concerns about the reliability of measurements, the process of measuring with an i.v. infusion line itself presents challenges. It typically requires at least two healthcare workers - one to hold and measure the line whilst the other performs the LP. Improvised approaches also increase the risk of procedural harms, especially in settings with limited procedural expertise [22]. Additionally, qualitative methods research has highlighted that a barrier for uptake by healthcare workers is a reluctance to use a method they perceive as inadequate or inferior to the gold standard [15]. The i.v. infusion line method might therefore be a disincentive to performing the procedure. Taken together, these challenges are reflected in the fact that, despite nearly universal access to i.v. infusion lines, the uptake of intracranial pressure management remains low.

### Is the use of manometers prohibitively expensive in low-resource settings?

Spinal manometers are often perceived as prohibitively expensive in low-resource settings, prompting the use of i.v. infusion lines as a lower-cost alternative. We considered the economic impact of adding manometers to the package of treatment using cost data from the AMBITION trial, a multi-country study conducted in five high-burden African countries [23]. In that 2020 analysis, the mean treatment cost per patient from the healthcare provider perspective was $1369, including the average cost of $29 for spinal manometers. Adjusted for inflation, this total equates to approximately $1680 in 2025 [24]. Within the trial participants received an average of six lumbar punctures (LPs), although two were conducted for trial specific outcome assessment. It is therefore reasonable to assume that four LPs per patient would reflect standard inpatient care.

Intravenous lines cost approximately $0.46 each ($1.84 per patient for four LPs) compared to approximately $5 each per manometer [25] ($20 per patient). This means spinal manometers may represent only 1.19% ($20/$1680) of total treatment costs. While i.v. lines are cheaper per unit, they require two clinical staff members to operate effectively and are less reliable for pressure measurement, potentially offsetting any perceived cost savings compared to manometers (Supplemental Data File appendix 1).

Within an exploratory analysis to evaluate the economic plausibility of spinal manometer use, we modelled the minimum mortality reduction required for a $20 per-patient investment to maintain the same cost per life saved (total treatment cost / survival). Using AMBITION-cm trial data, the average treatment cost per patient was $1680; subtracting the within-trial manometer cost of $29 yields a revised baseline of $1651. We assessed three 10-week mortality scenarios representing different hypothetical, clinically plausible care contexts without the use of manometers: 30% (controlled research settings [3,4]), 40% (routine care with optimized implementation [26]), and 50% (standard routine care [27,28]). Assuming untreated cryptococcal meningitis carries 100% mortality [28,29], the cost per life saved for meningitis treatment without manometers was $2359 ($1651/0.70), $2752 ($1651/0.60), and $3303 ($1651/0.50). To maintain these same ratios after including the $20 average cost for manometers per patient in routine care (raising the total to $1,671), the required absolute mortality reductions were minimal and likely under 1 percentage point across these plausible mortality scenarios (see Table 1 for detailed calculations).

**Table 1:** Threshold analysis of spinal manometer cost-effectiveness.

Scenario	Research setting			Optimized routine care			Standard routine care		
intervention	mortality rate	total cost	Cost per life saved	Mortality	Total cost	Cost per life saved	Mortality	Total cost	Cost per life saved
No treatment	1.00	0	NA	1.00	0	NA	1.00	0	NA
Baseline – total treatment cost without manometers	0.30	1651.35	2359.07	0.40	1651.35	2752.25	0.50	1651.35	3302.70
Intervention – total treatment cost with manometers	0.292	1671.35	2359.07	0.393	1671.35	2752.25	0.494	1671.35	3302.70

This model illustrates the absolute mortality reduction required to maintain the current cost-per-life-saved ratio (total treatment cost/mortality rate) when adding an estimated 20$ per-patient cost for spinal manometers. Baseline costs are derived from the AMBITION trial (adjusted to 2025 USD). Mortality scenarios represent varying care contexts: research (30%), optimized routine (40%), and standard routine (50%).

Spinal manometers therefore constitute a small fraction of overall treatment costs, and our findings suggest that only modest reductions in mortality are needed to economically justify their inclusion. Although the mortality reductions reported in observational datasets cannot be attributed solely to manometer use, they suggest that effective ICP management programs may achieve improvements substantially larger than the thresholds estimated here [13,14]. This modelling is only illustrative and does not incorporate real-world implementation challenges that go beyond simple unit-cost. The successful implementation of manometers depends on effective procurement pathways, regulatory barriers and supply chain issues, factors that are not captured in this unit-cost analysis. While comprehensive cost-effectiveness studies on approaches to the management of raised ICP in cryptococcal meningitis are still limited, the combination of potential mortality benefit and minimal additional cost supports wider adoption of manometers as a likely cost-effective intervention in routine care. Of note, wide adoption of manometers could also result in further enhancing this cost profile through economies of scale and unit cost reductions.

Cost alone is unlikely to be the sole reason for low uptake. Structural barriers such as fragmented procurement pathways, stock-outs of lumbar puncture kits, regulatory barriers to in-country device registration, classification by health services of manometers as non-essential devices, high staff turnover, competing clinical priorities, and limited procedural training also contribute. In many facilities, devices may not reach the bedside, not because they are unaffordable, but because they are not systematically bundled within essential procurement lists or implementation packages. Addressing these supply chain and institutional factors are likely to be as important as addressing the manometer unit price.

## Conclusion

Effective management of raised ICP is crucial for reducing mortality from cryptococcal meningitis and spinal manometers are the optimal device for both diagnostic and subsequent therapeutic LPs. While evidence on i.v. infusion lines as an alternative remains limited and conflicting, with the more robust evidence supporting a non-linear conversion between the two methods, they are preferable to having no pressure measurement when manometers are unavailable. Using published correction factors may mitigate some of the associated risks of pressure underestimation. Despite the widespread availability of i.v. infusion lines, ICP management remains underutilized in routine care due to barriers related to practice, training, and the perception of inferiority. Spinal manometers are the gold standard and improving their availability would support implementation of evidence-based ICP management and could improve care quality and outcomes. Emerging data on their cost-effectiveness and the relatively minor financial impact within overall care costs further support efforts to expand access.

As global HIV financing contracts, including the 2025 reductions in U.S. government support for HIV programs, ministries of health and implementing partners are increasingly required to prioritize core elements of care. In this context, identifying interventions that are both clinically important and economically plausible becomes critical. Spinal manometers are the most reliable tool for enabling accurate ICP measurement and are likely to be cost-effective particularly in low-resource settings where the impact of suboptimal ICP management is the most marked.

## Acknowledgements

Author contributions: Jonathan Falconer: principal manuscript drafting and revisions. Jack Adams: manuscript drafting and editing with focus on health economics analysis. Sile Molloy: manuscript reviewing and editing including senior review of economic analysis. Nelesh Govender: manuscript review and editing including senior review of scientific content. Joseph Jarvis: manuscript review and editing including senior review of scientific content. Thomas Harrison: manuscript review and editing including senior review of scientific content. David Lawrence: principal manuscript review, editing and senior review.

Funding sources: All authors were funded by the National Institute for Health and Care Research (NIHR134342; NIHR303140) **on HIV-associated Fungal Infections** using UK international development funding from the UK Government to support global health research. The views expressed in this publication are those of the authors and not necessarily those of the NIHR or the UK government.

Use of AI: ChatGPT version 4.0 utilized for simple language and grammar editing.

### Conflicts of interest

There are no conflicts of interest.

## Supplementary Material

Supplemental Digital Content
