# Supplementary material for: Under pressure: a call to increase access to spinal manometers as a critical component of the management of cryptococcal meningitis
Source: AIDS. 2026 Jul 30;40(9):1219–24. doi: 10.1097/QAD.0000000000004545 (PMC13566448; doi:10.1097/QAD.0000000000004545)
Supplement: Supplementary file 1 [file aids-40-1219-s001.docx]

Table 1. Threshold Analysis of Spinal Manometer Cost-Effectiveness. This model illustrates the absolute mortality reduction required to maintain the current cost-per-life-saved ratio (total treatment cost / mortality rate) when adding an estimated 20$ per-patient cost for spinal manometers. Baseline costs are derived from the AMBITION trial (adjusted to 2025 USD). Mortality scenarios represent varying care contexts: Research (30%), Optimized Routine (40%), and Standard Routine (50%).

| *Scenario* | *Research Setting* | | | *Optimized Routine Care* | | | *Standard Routine Care* | | |
| --- | --- | --- | --- | --- | --- | --- | --- | --- | --- |
| *Intervention* | Mortality rate | Total Cost | Cost per life saved | Mortality | Total Cost | Cost per life saved | Mortality | Total Cost | Cost per life saved |
| No treatment | 1.00 | 0 | NA | 1.00 | 0 | NA | 1.00 | 0 | NA |
| Baseline – total treatment cost without manometers | 0.30 | 1651.35 | 2,359.07 | 0.40 | 1651.35 | 2,752.25 | 0.50 | 1651.35 | 3,302.70 |
| Intervention – total treatment cost with manometers | 0.292 | 1,671.35 | 2,359.07 | 0.393 | 1,671.35 | 2,752.25 | 0.494 | 1,671.35 | 3,302.70 |
